# Chronic Granulomatous Disease (CGD): Commonly Associated Pathogens, Diagnosis and Treatment

**DOI:** 10.3390/microorganisms11092233

**Published:** 2023-09-05

**Authors:** Angel A. Justiz-Vaillant, Arlene Faye-Ann Williams-Persad, Rodolfo Arozarena-Fundora, Darren Gopaul, Sachin Soodeen, Odalis Asin-Milan, Reinand Thompson, Chandrashekhar Unakal, Patrick Eberechi Akpaka

**Affiliations:** 1 Department of Paraclinical Sciences, Faculty of Medical Sciences, The University of the West Indies, St. Augustine, Trinidad and Tobago; arlene.williams@sta.uwi.edu (A.F.-A.W.-P.); sachin.soodeen@my.uwi.edu (S.S.); reinand.thompson@sta.uwi.edu (R.T.); chandrashekhar.unakal@sta.uwi.edu (C.U.); patrick.akpaka@sta.uwi.edu (P.E.A.); 2 Eric Williams Medical Sciences Complex, North Central Regional Health Authority, Champs Fleurs, Trinidad and Tobago; rodolfo.fundora@sta.uwi.edu; 3 Department of Clinical and Surgical Sciences, Faculty of Medical Sciences, The University of the West Indies, St. Augustine, Trinidad and Tobago; 4 Department of Internal Medicine, Port of Spain General Hospital, The University of the West Indies, St. Augustine, Trinidad and Tobago; darren.gopaul2@my.uwi.edu; 5 Independent Researcher, Laval, QC H7E 2Z8, Canada; odalis_asin@yahoo.es

**Keywords:** chronic granulomatous disease, microorganisms, neutrophils, antimicrobials

## Abstract

Chronic granulomatous disease (CGD) is a primary immunodeficiency caused by a defect in the phagocytic function of the innate immune system owing to mutations in genes encoding the five subunits of the nicotinamide adenine dinucleotide phosphatase (NADPH) oxidase enzyme complex. This review aimed to provide a comprehensive approach to the pathogens associated with chronic granulomatous disease (CGD) and its management. Patients with CGD, often children, have recurrent life-threatening infections and may develop infectious or inflammatory complications. The most common microorganisms observed in the patients with CGD are *Staphylococcus aureus*, *Aspergillus* spp., *Candida* spp., *Nocardia* spp., *Burkholderia* spp., *Serratia* spp., and *Salmonella* spp. Antibacterial prophylaxis with trimethoprim-sulfamethoxazole, antifungal prophylaxis usually with itraconazole, and interferon gamma immunotherapy have been successfully used in reducing infection in CGD. Haematopoietic stem cell transplantation (HCT) have been successfully proven to be the treatment of choice in patients with CGD.

## 1. Introduction

**Figure 1:**
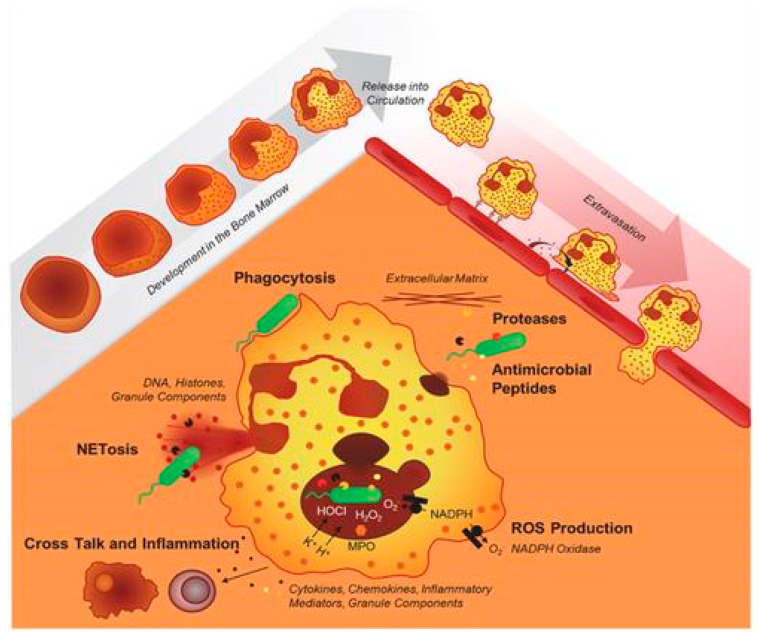
Schematic representation of normal phagocytic function reproduced from Kruger et al. (2015). Copyright: © 2015 Kruger et al. This is an open-access article distributed under the terms of the Creative Commons [8].

Chronic granulomatous disease (CGD) is a rare inherited primary immunodeficiency, that was first reported as a “Fatal granulomatous disease of childhood” owing to the early death of children with this condition, and has been described by some other authors [1,2,3,4,5,6,7]. A schematic representation of the neutrophil effector functions required to achieve an adequate primary immune defense has recently been described in this condition. It is displayed in reports by Kruger P et al. (Figure 1) [8]. Neutrophils develop in the bone marrow; band neutrophils are released into circulation and subsequently travel to tissues and organs to fight infections using various mechanisms, including phagocytosis, production of reactive oxygen species, and release of antimicrobial peptides, to destroy pathogens [8].

CGD is caused by the impaired phagocytic function of the innate immune system cells owing to mutations in genes encoding the five subunits of the nicotinamide adenine dinucleotide phosphate (NADPH) oxidase enzyme complex (OEC) [4]. The normal respiratory burst process is critical in killing pathogens, manifesting as CGD when it is dysfunctional.

This narrative literature review covers the most critical aspects of CGD in a comprehensive manner. The topics covered in this study include the pathogenesis, the clinical manifestations, and the incidence of CGD. In addition, it describes the granulomas, the pathogens, and the CGD-related infectious diseases, as well as the laboratory diagnosis and the management of CGD. At the end, a final figure gives a summary of the main findings that are reported in the conclusions.

## 2. Pathogenesis

The CGD’s inability to produce reactive oxygen species leads to pathognomonic systemic granuloma formation and increased susceptibility to recurrent and severe opportunistic bacterial and fungal infections. It causes unrestrained inflammation and autoimmunity. Since the condition was first described [1], there have been several improvements in treatment, such as antibacterial, antifungal, immunomodulatory, and hematopoietic stem cell transplantation, which extended the life expectancy of patients [9].

**Figure 2:**
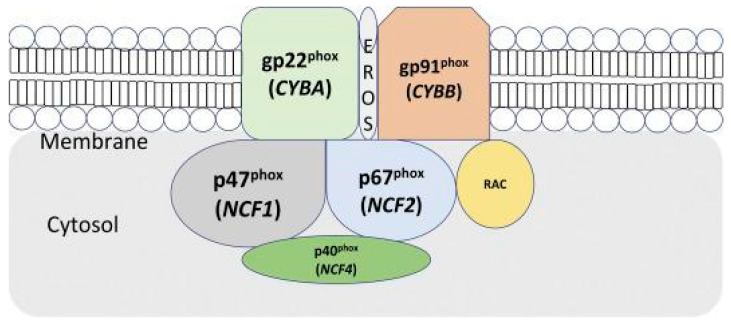
Illustration of the NADPH oxidase complex subunits (OEC). The membrane bound subunits gp22^phos^ (CYBA) and gp91^phox^ (CYBB) components also include the molecule for essential reactive oxygen species (EROS), which interacts with other proteins in the membrane. Adapted from Anjani et al. (2020) [4].

The severity of the phenotypes can vary depending on the mode of genotype inheritance, which is most severe in the X-linked type, followed by the autosomal recessive type [10]. The functional NADPH OEC comprises five subunits: two are localized in the cell membrane during the resting phase, and three are localized in the cytoplasm. The two membrane-bound subunits are gp91^phox^ and p22^phox^ (Figure 2). These proteins form a heterodimeric complex (cytochrome b558). The cell membrane’s contact with a pathogen activates the protein complex, and three cytoplasmic subunits (p47^phox^, p67^phox^, and p40^phox^) form a hetero-trimer translocating to cytochrome b558 [4].

Mutations in either the membrane or cytosolic domain disrupt the respiratory burst in phagocytes [11], as displayed in Figure 2 [9]. The clinical characteristics and rigour of the disease, as well as patient survival, depend significantly on the gene, type, and position of the mutation [4]. It was demonstrated for the first time that EROS (CYBC1/C17ORF62) regulates abundance of the gp91^phox^-p22^phox^ heterodimer of the phagocyte NADPH OEC in human cells. Essential reactive oxygen species (EROS) mutations are a novel cause of chronic granulomatous disease [6]. Encoding the p40^phox^ subunit of the phagocyte NADPH oxidase, has been described in only one patient. However, a report on 24 p40^phox^-CGD patients from families in eight countries exist. These individuals display eight different in-frame or out-of-frame mutations of p40^phox^ (NCF4), which are homozygous almost in the totality of the families [12].

## 3. Clinical Manifestations

Patients usually present with fever, malaise, or weight loss. Perirectal abscesses are also typical in patients with CGD and can persist for years despite aggressive antimicrobial treatments and intense local care. Several pathogens are associated with infections in CGD and these granulomas produce active lesions that in many cases are sterile with no pathogens involved. For example, in inflammatory bowel diseases (IBD), see below. In these circumstances, chronic inflammatory cell responses consisting of activated lymphocytes and histiocytes evolve and arrange to form granulomas, one of the hallmarks of CGD, provoking diverse clinical manifestations of obstruction such as delayed gastric emptying, antral narrowing of the stomach, dysphagia, emesis, weight loss, biliary tract or gastrointestinal obstruction [13].

### 3.1. CGD-Related Inflammatory Responses

There is an increased risk of autoimmune disorders such as inflammatory bowel colitis and inflammatory bowel disease among patients owing to increased activation of NF-kB, increasing the production of proinflammatory cytokines. The inflammatory manifestations of CGD are mainly observed in the GI and urogenital tracts, lungs, and eyes. Inflammation can be suppressed by blocking TNF-alpha and oral corticosteroids [14]. Immunomodulators for CGD-related inflammatory manifestations are under investigation, including pioglitazone, tamoxifen, and rapamycin [14].

### 3.2. Hemophagocytic Lymphohistiocytosis (HLH)

In addition, patients with CGD experience infection-triggered hemophagocytic lymphohistiocytosis (HLH), which presents as pathological hyperactive inflammation [15]. Possible pathologies, including CGD, should be considered in children with HLH because it can indicate CGD. An optimal management strategy is yet to be developed for children with CGD who manifest with HLH. Early recognition and proper management of infectious triggers and HLH are crucial to reducing mortality [3].

## 4. CGD Incidence

Pediatric CGD is relatively rare; this genetic condition, which has variable ethnic associations, occurs in 1 out of every 200,000–250,000 births in the United States and is often diagnosed in the first three years of life [9,16]. According to data from European nations, approximately 65% of patients with CGD have a molecular defect in *CYBB* (most are hemizygous males). Autosomal recessive CGD accounts for approximately 30% of all CGD cases. Molecular defects in any of these five genes (*CYBB* for gp91^phox^ (located on the X chromosome), *CYBA* for p22^phox^, *NCF1* for p47^phox^, *NCF2* for p67^phox^, and *NCF4* for p40^phox^) can occur in 90% of patients with CGD. They harbour mutations in the CYBB (gp91^phox^) or NCF1 (p47^phox^) genes [11]. Symptoms may be delayed in some patients with residual activity [15]. In most countries, the offspring of CGD consanguineous marriages or unions is at increased risk of CGD due to expressing autosomal recessive gene mutations inherited from common CGD ancestors. Therefore, consanguineous unions increase the incidence of this condition, which sometimes is not detected quickly enough, and death from infectious diseases may occur [17,18,19,20].

A large cohort study suggested that North African/Arab and Turkish immigrants in Europe have a high prevalence of autosomal recessive CGD, reflecting the increased prevalence of consanguineous marriage in these populations. The study proves that pulmonary sites occur more in number, which is well illustrated by this European study in the table below [21]. Approximately 1.49 per every 10,000 live births in the Israeli Arab population and 1.05 in the Israeli Jewish population have CGD, primarily associated with an autosomal recessive inheritance, displaying increased morbidity [20]. Late presentation of CGD has been reported [22]. CGD derives its name from the characteristic formation of multiple granulomas in various body tissues. Granulomas often occur anywhere in the GI tract, from the mouth to the anus, with the colon and oesophagus being the most and least common sites, respectively [23].

## 5. Granulomas

In patients with CGD, microgranulomas, tissue eosinophilia, and brown-pigmented epithelioid histiocytes found in the lamina propria and inflammatory changes revealed these distinctive features from biopsy materials [21,24]. Alimchandani et al. (2013) conducted a study on 87 patients with CGD and observed using GI biopsy that 74% (64/87) of patients had prominent brown granular cytoplasmic pigmented inclusions in macrophages [25]. Multiple aseptic granulomas most frequently form in the skin. Granulomas are active lesions and in many cases are sterile [21].

Inflammatory bowel disease (IBD) is becoming increasingly relevant in CGD [26,27]. Angelino et al. (2017) presented 9 out of 20 patients with CGD-IBD during diagnosis and/or follow-up. They mainly complained of nonspecific diarrhea (55%) and extensive colonic involvement (44%) [28]. Patients with CGD tend to have an abnormally excessive inflammatory response. These non-caseating granulomas affect the hollow viscera, most notably the stomach, colon, and bladder. These granulomas are likely unrelated to infections because microorganisms are usually not identified, and patients respond rapidly to steroids or other immunomodulators such as cyclosporine [27].

## 6. Pathogens and CGD-Related Infectious Diseases

### 6.1. Pathogens

Owing to changes and the introduction of new prophylactic treatments after the initial emergence of CGD, the median age of death has increased over the last few decades, with fungal infections being the highest risk of mortality [16,23]. The five most common pathogens that infect North American patients are *Staphylococcus aureus*, *Aspergillus* spp., *Nocardia* spp., *Burkholderia* spp., and *Serratia* spp. [23,24] in contrast to patients from Europe, where the five most common pathogens are *Staphylococcus aureus*, *Aspergillus*, *Salmonella*, *Candida*, and *Serratia species*. The infections can be severe or opportunistic and unusual because of fungi and bacteria that cause suppurative lymphadenitis, pneumonia, and abscesses at various locations [19,23]. These are also discussed below regarding signs, symptoms, and complications.

Chronic Granulomatous Disease patients are susceptible to a subset of catalase-positive organisms (CPO) because CPO degrades host-produced hydrogen peroxide before its conversion to hypochlorous acid by myeloperoxidase [9]. These CPOs include bacteria such as *Pseudomonas* spp., and *Enterobacteriaceae* such as *Klebsiella* spp. [16,21,23].

Fungi including *Neosartorya udagawae,* and *Sporothrix schenckii* have been reported in patients with CGD treated with antifungal prophylaxis [24]. Other rare pathogens that have been cited, including sporadic cases of *Cephalosporium*, *Streptococcus pneumoniae*, *Scedosporium*, *Paecilomyces aecilomyce*, *Staphylococcus epidermidis*, *Rhodococcus equi* and *Phialophora richardsiae* [21]. Non-Aspergillus fungal infections are prevalent and can be associated with *Rhizopus* spp. and *Trichosporon* spp. They were reported in nine cases and the lung was the most commonly affected organ [26]. In addition, other fungi that are not Aspergillus species have emerged in the era of mould prophylaxis [29]. Of these, *Phellinus* spp. can cause invasive fungal infections (IFIs) in CGD [27]. Two species have been recognised *Phellinus tropicalis* and the basidiomycete *Phellinus mori* [30].

### 6.2. CGD-Related Infectious Diseases

Notably, severe recurrent bacterial and fungal infections usually present early in childhood (<5 years of age) in most patients with CGD. This is attributed to severe respiratory burst defects and the lack of EROS production. However, symptoms are delayed until adolescence and adulthood owing to the degree of residual NADPH oxidase activity [31].

The most frequent fungal infection is invasive aspergillosis caused by *Aspergillus fumigatus*, followed by *Aspergillus nidulans* and less common *Aspergillus terreus*, which has been isolated from bronchoalveolar lavage of patients with CGD [16,32]. Other reported species include *Aspergillus niger* and *Aspergillus tanneri* [33]. Fungal infections, particularly those caused by *Aspergillus* spp., are significant determinants of morbidity and the most common cause of mortality in patients with CGD [16,20]. The most common sites of infection are the lungs, followed by the skin, lymph nodes, liver, and gastrointestinal tract [21].

#### 6.2.1. Lung Involvement

Pneumonia is the most common pulmonary disease reported in patients with CGD, caused primarily by *Aspergillus* spp. and *Staphylococcus aureus* [29]. Microbial pneumonia differs from mulch pneumonitis which typically develops within seven to ten days of exposure to organic materials including mulch, wood chips, hay or leaves. The mortality rate for mulch pneumonitis in a case series of patients with chronic granulomatous disease was more than 50% [34]. Mulch pneumonitis is a medical emergency and should be considered in all cases of unexplained pneumonitis, particularly in patients with acute onset and hypoxia. These patients should be treated with high-dose corticosteroids and antifungal and antibacterial agents [35,36,37]. Lung abscesses are relatively less common but potentially severe [16,21,23,31].

Mycobacterial infections caused by Bacillus Calmette–Guérin (BCG; in endemic countries that routinely administer vaccines) and *Mycobacterium tuberculosis* have been reported in Israel, Turkey, Iran, China, and Latin America. However, these patients present with a more localized disease [35,36,37].

#### 6.2.2. Skin Site Involvement

Subcutaneous abscesses are the most common, frequently located in the perianal region. They are typically caused by *S. aureus* but can also be caused by Serratia spp., *Aspergillus* spp., and *Klebsiella* spp. [21,23].

Suppurative or necrotising lymphadenitis is common in patients with CGD. Individuals with autosomal recessive forms of CGD have a lower probability of suppurative lymphadenitis than those with the X-linked form, suggesting that residual oxidase, which is more frequent in patients with the autosomal recessive disease, might enhance protection against this complication [38]. Suppurative lymphadenitis can also result from region-specific medical practices. Patients with CGD are predisposed to lymphadenitis after receiving BCG vaccination; however, their disease is rarely disseminated [39]. Frequent and chronic infections have consequences; patients with CGD reportedly present with a failure to improve owing to the long-term use of treatments [40]. Cellulitis was also reported by Winkelstein et al. (2000), although it is relatively rare [23].

#### 6.2.3. Liver Involvement

Liver abscesses are a frequent complication in patients with CGD and can cause significant morbidity. However, the signs and symptoms of this complication are variable and nonspecific, with the most common being fever, malaise, weight loss, abdominal tenderness, and elevated erythrocyte sedimentation rate [41]. In cases with liver abscesses, the predominant organism isolated was *Staphylococcus aureus*. Liver involvement in patients with CGD is a notable concern because splenomegaly, nodular regenerative hyperplasia, non-cirrhotic portal hypertension, and portal venopathy can occur [42]. Splenomegaly can subsequently cause thrombocytopenia, which has been reported to be a poor prognostic indicator in patients with CGD [43].

#### 6.2.4. Gastrointestinal Tract Involvement

The gastrointestinal tract symptoms in patients with CGD are generally nonspecific and range from mild to debilitating symptoms, such as abdominal pain, bloody diarrhoea, nausea, vomiting, malabsorption, and weight loss [44]. The rate of GIT involvement is much higher in the X-linked than in the autosomal recessive form, as reported in an extensive survey of patients with CGD conducted by Marciano et al. [39]. The GIT involvement, particularly inflammatory bowel disease (IBD), could be the first sign of undiagnosed CGD [25].

#### 6.2.5. Bone Involvement

In patients with CGD, osteomyelitis occurs in males aged 4–20 years. X-linked inheritance has been reported in nine patients with osteomyelitis caused by *Aspergillus nidulans.* Conversely, among three patients with *Aspergillus fumigatus* infection, osteomyelitis was associated with X-linked gp91^phox^ in two patients and the autosomal recessive form of p67^phox^ in one [38].

#### 6.2.6. Other Involvements

An interesting data is that children with CGD had predominantly mild infection with COVID-19 among a cohort of 101 CGD patients [45]. However, CGD patients have normal immunity to most viruses. Two patients with severe COVID-19 have been reported in the literature, which suggests that COVID-19 might have a different pathogenesis than other viruses [46].

In a multicenter collaborative study of CGD in India, where there were investigations of 236 patients, X-linked and AR-CGD were seen in 77 and 97, respectively [47]. Table 1 depicts the different sites of infections in patients with CGD.

## 7. Laboratory Diagnosis

### 7.1. Neutrophil-Function Testing

Patients suspected of suffering from CGD are diagnosed by the inability of their blood phagocytes to generate reactive oxygen species [5]. Initial diagnostic tests for CGD often rely on the various measurements of neutrophil superoxide production [48]. These include (a) direct measurement of superoxide production [49], (b) Cytochrome C Reduction Assay—This is based on a colorimetric assay that measures the reduction of cytochrome C by NADPH-Cytochrome C reductase in the presence of NADPH. The reduction of cytochrome C results in the formation of distinct bands in the absorption spectrum and the increase in absorbance at 550 nm is measured with time [50,51], (c) Nitroblue tetrazolium (NBT) reduction test—The nitroblue tetrazolium reduction test (NBT) is an assay based on the activation percentage of neutrophils in peripheral blood. It has been used to study the follow-up of microbial agents owing to the narrow relationship between the molecules involved in the oxidative burst and the organisms e.g., Leishmania activity in phagocytes [51,52,53,54,55,56,57], (d) Dihydrorhodamine (DHR) 123 Oxidation test—White blood cells are incubated with dihydrorhodamine 123 (DHR) and catalase, then stimulated with Phorbol 12-Myristate 13-Acetate (PMA). Dihydrorhodamine oxidation to rhodamine by the respiratory burst of the cell is measured by flow cytometry [53,58] and (e) Chemiluminescence [58,59].

### 7.2. Nitroblue Tetrazolium (NBT) Reduction Test

The oldest laboratory test for CGD is the NBT test, often the primary screening test for CGD [60]. This test uses light microscopy to provide a rapid but relatively qualitative analysis of phagocyte NADPH oxidase activity. Superoxide produced by normal peripheral blood neutrophils reduces yellow NBT to dark blue/black formazan, which forms a precipitate in the cells [61]. The test is performed on a microscope slide, to manually distinguish reducing (blue-black) from non-reducing (unstained) cells manually. Normal phagocyte oxidase activity will result in at least 95% positive cells in this assay, while CGD patients will have absent or severely reduced production of superoxide and therefore minimally reduced cells [60]. Test limitations include a higher rate of false-negative results and operator subjectivity. This assay should be run in conjunction with blood from healthy control subjects to identify problems with specimen handling [62].

### 7.3. Flow Cytometric Dihydrorhodamine Assay

Dihydrorhodamine (DHR) assay testing is often the test of choice in diagnosing CGD [63]. DHR123 is a lipophilic nonfluorescent molecule that readily diffuses across cell membranes and localises in the mitochondria [50]. The molecule is oxidised to rhodamine 123 in stimulated phagocytes of the nicotinamide adenine dinucleotide phosphate (NADPH) oxidase and is trapped within cells in this form [51]. The quantitative nature of this assay allows for differentiation between oxidase-positive and oxidase-negative phagocyte CGD carriers [48] and diagnosis of gp91^phox^ and p47^phox^ deficiencies [52]. DHR also allows for measuring residual superoxide production and thus provides prognostic information for CGD patients [53]. DHR assays are relatively easier to perform, more reliable, more quantitative, and more sensitive when compared to NBT [58]. DHR assays can distinguish between X-linked and autosomal variants of CGD and detect gp91^phox^ carriers [59,64]. The DHR test can also be used to determine chimerism status following hematopoietic cell transplantation [65] which is important to evaluate for early engraftment and graft failure and thus, guides for early intervention [66]. Reactive oxygen species (ROS) production by neutrophils and monocytes can be analyzed by incubating whole blood with dihydrorhodamine 123 (DHR) and catalase in the presence or absence of phorbol 12 myristate 13 acetate (PMA) stimulation. Analysis can be performed on a BD FACS Canto II (flow cytometer). Fluorescence is based on absorption of photons with the concomitant emission of light [67].

### 7.4. Luminol-Enhanced Chemiluminescence Assay

Chemiluminescence is the emission of light (luminescence) as the result of a chemical reaction (e.g., oxidation). It is essentially an oxyluminescence since molecular oxygen is necessary for the reaction. Luminol is widely used to detect reactive oxygen species produced in biological systems and is used in CGD patients to measure ROS production [68].

### 7.5. Genetic Testing

The diagnosis of CGD based on positive abnormal neutrophil function testing should be followed by genetic testing for confirmation. Sequencing of the patient’s phagocyte oxidase (phox) genes is done to determine the exact molecular defect [48]. The most common p47^phox^ pathogenic defect in CGD is due to a pseudogene conversion and may be missed by standard sequencing [69]. Immunoblot (standard immunoblotting of neutrophils stained with phox-specific antibodies) or gene dose determination may be needed to confirm p47^phox^ deficiency [49]. Pathogenic variants in the CYBB gene encoding gp91^phox^ are mostly due to missense or nonsense mutations but can also be due to promoter, insertion, deletion or splice site mutations [70,71]. Nonsense variants generally lead to more severe and fatal CGD [5]. Missense mutations identified by gene testing can be associated with residual superoxide formation, some DHR positivity, and better survival, inhibit critical protein functional domains to complete loss of DHR activity and lead to more severe CGD with diminished survival, depending on the amino acid sequence affected [53]. Thus, gene sequencing prerogative prognosis, mortality risk allow for counseling on bone marrow transplantation or gene therapy.

Other techniques used in genetic testing include the expression pattern of different NADPH components by flow cytometry as a screening tool to identify the underlying affected gene, next-generation sequencing (NGS), Sanger sequencing and Gene scan analysis [70,72].

## 8. Management of CGD

### 8.1. Haematopoietic Stem Cell Transplantation (HSCT/HCT)

HCT is the principal treatment for managing CGD with favourable results regardless of symptoms, age, sex, or mutations [73,74,75]. Transplantation therapy has an overall survival rate of over 90% in children under 14 years and has improved in the last decade, particularly with early diagnosis [74]. Additionally, HCT is associated with event-free survival rates of more than 80% in patients with CGD and improves the quality of life [76]. There are debates about designing optimal conditioning protocols using myeloablative or reduced-intensity regimens [14].

However, the group of HCT-treated patients demonstrated excellent survival rates, although the risks and benefits still need to be assessed in individual patients. Based on the significant progress of patients with CGD treated with HCT, it is regarded as the only known curative treatment with an improved life expectancy owing to its improved implementation over time [77,78,79,80].

When Human Leucocyte Antigen (HLA)-matched donor is identified, the source of HCT could be cord blood, bone marrow, or peripheral blood [75]. Hematopoietic stem cells are drawn and infused into the patient. These are immature cells, and after they develop into platelets, red- and white-blood cells. A range from 70–95% is proven as survival rate for this type of immunotherapy. Several factors affect the outcome of HCT, including the age and clinical status of the child. As earlier HCT would improve its clinical outcome. The patient must be subjected to various chemotherapy treatments. It is done to equip the patient’s immune system to receive new stem cells. In addition, locating HLA-compatible donors is time-consuming, and the child with CGD must be adequately treated to prevent worsening infections [81,82]

### 8.2. Drug-Based Treatment

Antimicrobial and antifungal prophylaxis are the most common management routes used to minimize the incidence of infections. However, treatment with antibiotics is contraindicated in healthy patients because of antibiotic resistance. Most studies suggest a link between aggressive antibiotic use and preventing the spread of infection in patients with CGD [83].

Drugs such as trimethoprim-sulfamethoxazole reduce the occurrence of bacterial infections in patients with CGD but do not interfere considerably with the gut microbiome [37]. Patients with sulfamethoxazole allergy have other options, such as cloxacillin and ciprofloxacin [74]. A concern arises in pregnancy since trimethoprim is a folic acid antagonist, which increases the high risk for congenital disabilities and is discontinued during pregnancy [73]. Itraconazole considerably reduces invasive fungal infections, and newer azole drugs, such as voriconazole, posaconazole, and isavuconazole, are available, providing more options for treating these fungal infections [73].

Itraconazole should be provided as a long-term and possible lifelong treatment option to prevent fungal infections in children with CGD. However, regular monitoring of liver function is required during itraconazole therapy [37]. In cases where patients are intolerant to itraconazole, posaconazole is a safer and more effective option [74].

In patients with CGD, bacterial infections are commonly caused by *Staphylococcus aureus*, *Aspergillus* spp., *Nocardia* spp., *Burkholderia* spp., and *Serratia* spp. [84] and several antimicrobials have been therapeutically used [13,85,86].

CGD treatment should start at the earliest, and before the microbiological cultures are available. Antimicrobials should be given parenterally. Bacterial infections such as *S. aureus* and gram-negative bacteria, including *B. cepacia* complex, can be treated with a combination of ceftazidime and nafcillin and or a carbapenem. However, *Burkholderia* is typically resistant to most aminoglycosides. If the infection persists for 24–48 h, then more diagnostic tests should be done to identify the responsible microorganism. Additional antibiotic coverage such as high-dose intravenous trimethoprim-sulfamethoxazole to cover ceftazidime-resistant *B. cepacia* and *Nocardia* should be available [87,88].

If fungus is identified, antifungal treatment should be institutionalised even before the diagnosis is confirmed. Lung and bone aspergillosis are very prevalent and require prolonged therapy. The echinocandin antifungals including micafungin, caspofungin, and anidulafungin can effectively treat refractory *Aspergillosis* in patients unresponsive to lipid-formulated amphotericin B and azoles. Intravenous antifungals must be early considered in CGD patients [89].

Treatment using TNF-alpha inhibitors in patients with CGD could help improve the outcome of severe inflammatory complications despite the associated risk factors. This treatment could provide short-term benefits in selected patients with CGD with severe inflammatory complications awaiting HCT [90]. There is conflicting evidence regarding infliximab, a TNF-alpha inhibitor, causing rapid improvement; however, it is associated with an increased risk of severe infections and death in patients with CGD and should be strictly avoided. It is owing to a study involving five patients [74,91]. In addition, corticosteroid use has proven beneficial for CGD colitis; however, their use has traditionally been contraindicated in patients with CGD and active infection. In conjunction with appropriate antimicrobials, steroids help treat hyperactive inflammatory responses [73,74]. Corticosteroids, despite their effectiveness, are associated with long-term complications such as growth retardation, osteoporosis, and an increased risk of infection [41,92,93,94,95].

To determine the optimal treatment for patients with CGD, a European study compared conventional treatments with HCT. Some patients under conventional treatment did not improve. Seventy-six per cent (76%) of these patients were affected by inflammatory complications, whereas 85% developed at least one infection even with conventional treatments, the most common being skin infection and pneumonia [87,88,96,97,98,99,100].

For inflammatory conditions, steroid treatment with immunosuppressants (such as anti-tumour necrosis factor) is adequate as second-line therapy, as they exhibit some efficacy. However, immunosuppressant (such as anti-tumor necrosis factor agents, thalidomide, and anakinra) use is still debated because of its risks, notwithstanding its benefits [14,101,102].

Lugo-Reyes et al. (2022) reported the outcomes of a systematic review and meta-analysis on IFN-γ’s efficacy and safety in CGD [103]. They support the use of IFN-γ in managing patients with CGD. However, the authors did not find sufficient clinical evidence and suggested that more clinical trials are needed to assess the efficacy and long-term safety of IFN-γ. As the longevity of patients with CGD improves, a long-term and detailed assessment of the autoimmune and inflammatory complications associated with chronic IFN-γ therapy is required. For the clinicians whose patients continue to die during adolescence owing to invasive pulmonary aspergillosis, especially in Latin America, The Caribbean and other regions where resources are scant, it is imperative to ascertain the patients who are prescribed long-term use of IFN-γ and also identify the significant risks for complications [103].

### 8.3. Gene Therapy

Gene therapy remains in the experimental stage. A recent human trial involved nine patients with X-linked CGD undergoing ex-vivo autologous CD34+ haematopoietic stem-and progenitor cell-based lentiviral gene therapy following myeloablative conditioning. Two of the nine patients died during the trial; however, prophylactic antibiotic treatment was no longer required in the surviving patients. Moreover, stable vector copy numbers and no clonal dysregulation or transgene silencing were identified in six surviving patients with CGD [84,104,105,106].

Current gene therapy trials, which remains experimental, have demonstrated that lentiviruses or gene editing can be used as curative therapy where HCT is inappropriate for a patient and removes the risk of graft-versus-host disease. Notably, in the future, gene therapy could be applied when human leukocyte antigen (HLA)-matched donors are difficult to identify, and HCT is not feasible. It is a promising method that involves the insertion of a functional copy of a gene into the correct cells, where success depends on viral vectors. Lentiviral systems are currently the main techniques used to deliver therapeutic genes in experimental gene therapy for treating CGD [107]. These advances in gene therapy have facilitated more accurate treatment procedures [108]. Furthermore, gene therapy as a cure for CGD is a crucial area of research, specifically for patients with X-linked and p47 mutations [109].

There are promising future approaches for treating patients with CGD, including genome-editing technologies, such as CRISPR/Cas9 nuclease gene therapy [85,110] and SIN-lentiviral vectors. A multicenter trial is currently being carried out in the United States and Europe to determine the feasibility of gene therapy for patients with CGD [74]. Other authors have experimentally reported the use of gene therapy in CGD patients. It could provide life-saving clinical benefit to CGD patients lacking a suitable donor [111,112].

### 8.4. Other Therapies

CGD defects revealed that the direct repair of the defect in CGD could be performed using thymosin β4 subverts. “Tβ4 promotes HIF-1α stabilization and, in turn, HIF-1α may transcriptionally regulate Tβ4 expression”. It is in experimental stage and is not a type of gene therapy. Hypothetically, thymosin β4 was seen to restore the body’s ability to remove damaged cells and renew healthier cells in patients with CGD by restoring autophagy and upregulating hypoxia-responsive genes in human and murine CGD. Autophagy, which may help in pathogen elimination, could prevent granuloma formation, commonly seen in CGD [108,113]. CGD leads to infections of the liver, lungs, and lymph nodes, and treatment of CGD with prophylactic drugs could be prolonged; therefore, optimal therapy must be chosen for these patients [33,82].

**Figure 3:**
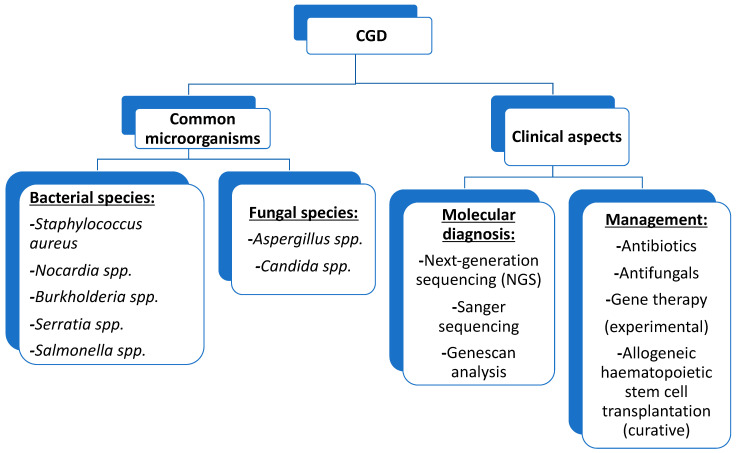
Summary of conclusions, including common microorganisms and clinical aspects.

Results from cases where multiple granulocyte infusion was performed, although not yet evaluated in controlled studies, suggest its usefulness in treating severe bacterial and fungal infections. Adverse effects, although well tolerated, include fever, developing leucoagglutinin, and rarely pulmonary leukocytosis [73]. Figure 3 summarises some of the most important aspects of this revision.

## 9. Conclusions

In conclusion, CGD is relatively rare, the most common microorganisms observed in patients with CGD are *Staphylococcus aureus, Aspergillus* spp., *Candida* spp., *Nocardia* spp., *Burkholderia* spp., *Serratia* spp., and *Salmonella* spp. Granulomas are active lesions, and in many cases are sterile. They provoke diverse clinical manifestations of obstruction such as delayed gastric emptying, antral narrowing of the stomach, dysphagia, emesis, weight loss, biliary tract, or gastrointestinal obstruction. The laboratory diagnosis of CGD includes state of the art techniques to measure ROS production in neutrophils and the detection of anomalies in the genome of CGD patients by testing, including the expression pattern of different NADPH components by flow cytometry as a screening tool to identify the underlying affected gene, next-generation sequencing (NGS), Sanger sequencing and Genescan analysis. The current management of patients with CGD involves a comprehensive multidisciplinary approach and its potential complications. Antibiotics and antifungals were once considered the most important treatment options for managing CGD. Despite starting as an experimental option, they helped achieve a high curative rate and longer life expectancy. Gene therapy may be considered an option to improve treatment outcomes but remains experimental. Although it initially led to clinical improvement, methylation of the viral promoter causes transgene silencing over time and the loss of therapeutic benefit.

Therefore, the treatment of CGD is progressing, from antibiotic prophylaxis developed in the 1970s to the current application of allogeneic haematopoietic stem cell transplantation [114] from a human leukocyte antigen-identical donor has been proven to cure CGD.

## Figures and Tables

**Table 1:** Site of disease.

Site of Disease	Number of Episodes	Number of Patients with ≥1 Episode	% of Patients with ≥1 Episode
Lung	634	284	66%
Skin/Subcutis	341	299	53%
Lymph node	622	213	50%
Gastro-intestinal	643	208	48%
Liver	240	138	32%
Kidney/ Urinary tract	139	95	22%
Septicaemia	111	85	20%
Ear	84	62	14%
Bone	84	56	13%
Eye	68	46	11%
Joint	35	31	7%
Brain	34	31	7%
Autoimmunity-Rheumatology	26	26	6%

Adapted from Van den Berg et al. (2009) [21].

## Data Availability

Not applicable.
